# Highly efficient TiO_2_-supported Co–Cu catalysts for conversion of glycerol to 1,2-propanediol

**DOI:** 10.1038/s41598-021-02416-7

**Published:** 2021-11-29

**Authors:** Wongsaphat Mondach, Sarun Chanklang, Pooripong Somchuea, Thongthai Witoon, Metta Chareonpanich, Kajornsak Faungnawakij, Hiesang Sohn, Anusorn Seubsai

**Affiliations:** 1 Department of Chemical Engineering, Faculty of Engineering, Kasetsart University, Bangkok, 10900 Thailand; 2 Center of Excellence on Petrochemical and Materials Technology, Kasetsart University, Bangkok, 10900 Thailand; 3 Research Network of NANOTEC–KU on NanoCatalysts and NanoMaterials for Sustainable Energy and Environment, Kasetsart University, Bangkok, 10900 Thailand; 4 Nanomaterials for Energy and Catalysis Laboratory, National Nanotechnology Center (NANOTEC), National Science and Technology Development Agency (NSTDA), Klong Laung, Pathumthani, 12120 Thailand; 5 Department of Chemical Engineering, Kwangwoon University, Seoul, 01897 Korea

**Keywords:** Chemistry, Chemical engineering, Engineering, Chemical engineering

## Abstract

Glycerol is a low-cost byproduct of the biodiesel manufacturing process, which can be used to synthesize various value-added chemicals. Among them, 1,2-propanediol (1,2-PDO) is of great interest because it can be used as an intermediate and additive in many applications. This work investigated the hydrogenolysis of glycerol to 1,2-PDO over Co–Cu bimetallic catalysts supported on TiO_2_ (denoted as CoCu/TiO_2_) in aqueous media. The catalysts were prepared using the co-impregnation method and their physicochemical properties were characterized using several techniques. The addition of appropriate Cu increased the glycerol conversion and the 1,2-PDO yield. The highest 1,2-PDO yield was achieved over a 15Co0.5Cu/TiO_2_ catalyst at 69.5% (glycerol conversion of 95.2% and 1,2-PDO selectivity of 73.0%). In the study on the effects of operating conditions, increasing the reaction temperature, initial pressure, and reaction time increased the glycerol conversion but decreased the selectivity to 1,2-PDO due to the degradation of formed 1,2-PDO to lower alcohols (1-propanol and 2-propanol). The reaction conditions to obtain the maximum 1,2-PDO yield were a catalyst-to-glycerol ratio of 0.028, a reaction temperature of 250 °C, an initial H_2_ pressure of 4 MPa, and a reaction time of 4 h.

## Introduction

During the last decades, many research topics have focused on the development of renewable and sustainable energy resources owing primarily to the restricted availability of petroleum reserves and growing environmental concerns. Biomass and biomass-derived chemicals have attracted attention because as renewable sources, they can replace petroleum-derived fuels for sustainable development. Biodiesel is one of the most widely used renewable fuels, which can be used to replace conventional petroleum-based diesel directly for both stationary and mobile applications. In biodiesel production via transesterification of triglycerides in fat or oil, approximately 10% of crude glycerol is produced as a by-product^1^. Furthermore, the rapid expansion of biodiesel production has led to a rapid increase of byproduct glycerol and a decrease in its price. Therefore, the procedure for generating high value-added products from glycerol has received considerable attention and will substantially improve the economic viability of biodiesel production.

A variety of valorizations of glycerol, such as selective oxidation, hydrogenolysis, dehydration, transesterification, reforming, and condensation over heterogeneous catalysts have been reported for converting glycerol into valuable products^1–5^. Among the various possibilities of glycerol valorization, the catalytic hydrogenolysis of glycerol to 1,2-propanediol (1,2-PDO) has been one of the attractive alternatives because 1,2-PDO is a non-toxic chemical and is widely used as an intermediate and additive in many applications such as polyester resins, anti-freezing, engine cooling, paints, cosmetics, pharmaceuticals, and foods^6–8^. Currently, 1,2-PDO is produced mainly via the selective oxidation of petroleum-derived propylene to propylene oxide followed by hydrolysis^9^. However, the fluctuation in petroleum cost and increasing environmental concern have severely affected the production of 1,2-PDO via petroleum-derivatives. On the other hand, 1,2-PDO is produced using acid-catalyzed dehydration of the primary hydroxy group of glycerol to generate acetol, followed by hydrogenation over a catalyst. The latter process is more environmentally sustainable.

Generally, hydrogenolysis is a class of reduction reaction that involves cleavage of the C–O bond in an organic compound with the simultaneous addition of a hydrogen atom to the resulting molecular fragments^8^. Specifically, the hydrogenolysis of glycerol to propanediol preferentially occurs over a bifunctional solid catalyst in either the vapor phase or the liquid phase of the reactants (glycerol and hydrogen). In other words, the catalyst for the hydrogenolysis of glycerol to 1,2-PDO must be bifunctional, with the first function being the dehydration at one of the primary hydroxyl groups of glycerol to hydroxy acetone (HA) and the second the hydrogenation of HA to 1,2-PDO as the product^10^. The catalysts shown to be active for the hydrogenolysis of glycerol to 1,2-PDO can be divided into two groups: (1) noble metal catalysts (such as Pt, Ru, Rh, Pd, and Re) are highly active in the activation of the hydrogen molecule; nevertheless, they often promote excessive cleavage of C–C bonds^11^ and more importantly, they are expensive; (2) transition metal catalysts (such as Cu, Ni, and Co) have been intensively investigated in the hydrogenolysis of glycerol because they are inexpensive and highly resistant to catalyst poisoning. Among those various active metal catalysts, Co-based catalysts are interesting for an oxidation–reduction reaction, because they exhibit very low C–C bond cleavage but high activity for the selective catalytic hydrogenation of alkenes, aldehydes, and ketones^12^. These catalytic properties are beneficial for the hydrogenolysis of glycerol to propanediols. Nevertheless, only a few reports on Co-based catalysts have been reported in the hydrogenolysis of glycerol. For example, Gou et al. studied bi-functional Co/MgO catalysts, where the interaction between Co species and MgO was adjusted by the temperature of calcination for the glycerol hydrogenolysis to 1,2-PDO at 473 K, 2.0 MPa, and 9 h. They found that calcination at 873 K improved the interaction between Co_3_O_4_ and MgO, resulting in the prevention of aggregation of Co particles in the catalyst under extreme conditions. Their optimal glycerol conversion was 44.8% with 42.2% selectivity of 1,2-PDO^13^. Rekha et al. reported on a series of Co-ZnO catalysts prepared using the co-precipitation method and varying the Co-to-Zn ratio for the hydrogenolysis of glycerol to 1,2-PDO. The activity of the catalysts depended on the weight ratio of Co and ZnO and the maximum catalytic activity was obtained with a 50:50 weight ratio of Co-to-ZnO, with 70% glycerol conversion and 80% selectivity of 1,2-PDO^14^.

The hydrogenolysis of glycerol also preferentially requires acidic sites from the catalysts because acidic sites can promote the dehydration of glycerol to HA, which is subsequently transformed to 1,2-PDO via hydrogenation^11,13,14^. In this manner, a catalyst support is commonly used to provide catalyst acidity. Thus, the selection of the catalyst support in the hydrogenolysis of glycerol to 1,2-PDO is also crucial. Various catalyst supports have been widely studied for the hydrogenolysis of glycerol, such as Al_2_O_3_, SiO_2_, ZnO, TiO_2_, and zeolite. Nevertheless, little research has been reported on the use of TiO_2_ as an acidic support for the hydrogenolysis of glycerol. For example, Feng et al. studied the effect of support for the hydrogenolysis of glycerol. They found that the TiO_2_ support had the smallest metal particle sites, resulting in the highest activity in the hydrogenolysis of glycerol. The maximum glycerol conversion was 90.1% with 20.6% selectivity of 1,2-PDO^15^. Salazar et al. reported that the 2.5Ru-2.5Cu/TiO_2_ catalyst had the best performance of the hydrogenolysis of glycerol to 1,2-PDO because TiO_2_ produced the smallest and most stable Ru particles, obtaining the highest yield of 1,2-PDO at 6.9% with 10% glycerol conversion and 69% selectivity of 1,2-PDO^16^.

In the current study, we report for the first time the synthesis of bimetallic catalysts of Co and Cu supported on TiO_2_ and their use for the hydrogenolysis of glycerol to 1,2-PDO. The characterization of the catalysts was also be studied to determine the chemical-physical properties, which were used to correlate with catalyst performance. Furthermore, the effect of the operating conditions—catalyst-to-glycerol ratio, reaction temperature, initial reaction pressure, and reaction time—was investigated for the hydrogenolysis of glycerol. Finally, the reusability of the catalyst was studied to determine catalyst stability after use for several cycles.

## Materials and methods

### Preparation of catalysts

The TiO_2_ (21 nm primary particle size (TEM), ≥ 99.5% trace metals, Sigma-Aldrich) was used as a catalyst support. The CoCu/TiO_2_ catalysts were prepared using the co-impregnation method. Aqueous solutions of Co(NO_3_)_2_•6H_2_O (2 M, 98%, QReC) and Cu(NO_3_)_2_•3H_2_O (1 M, 99%, Ajax) were used as the precursors for Co and Cu, respectively. The amount of Co was kept at 15 weight percent (wt%) but the amounts of Cu were varied at 0, 0.25, 0.5, 1.0, or 2.0 wt.%. The predetermined amount of the Co and Cu solutions was loaded on the TiO_2_ support. Then, each mixture solution was continuously stirred at room temperature for 1 h. The mixture was then dried in a hot-air oven at 100 °C for 12 h and calcined in an air furnace at 500 °C and a heating rate of 5 °C min^-1^ for 4 h to obtain xCoyCu/TiO_2_ catalysts (where x = 15 wt% of Co and y = 0, 0.25, 0.5, 1 or 2 wt% of Cu, respectively).

### Catalyst activity studies

The activity of each prepared catalyst was evaluated in a teflon-lined stainless-steel autoclave (Parr 4848, actual volume = 200 mL), which was equipped with an electromagnetic stirrer and a temperature controller unit. Before the reaction, each catalyst was reduced using H_2_ at a flow rate of 50 mL min^−1^ at 350 °C for 1 h in a plug flow reactor. Then, the catalyst was transferred to the teflon-lined stainless-steel reactor. Subsequently, glycerol (10 mL, 99.5%, QReC) and deionized water (10 mL) were rapidly introduced into the reactor to prevent the reduced catalyst from contact with air. Afterward, the reactor was sealed and purged three times with pure H_2_ (99.999%, Air liquide) to eliminate any air. The reactor was pressurized to the desired H_2_ pressure (2, 4, or 6 MPa) and the stirring speed was set at 800 rounds per minute (rpm). Then, the reactor system was heated to the desired reaction temperature (210, 230, 250, or 270 °C). After the reaction, the reactor was cooled to room temperature and the pressure was released to ambient conditions. A small amount of the liquid products (5.0 mL) was then sampled using a syringe filter (nylon 0.45 µm, CNW Technologies) for product analysis using gas chromatography (Shimadzu, GC-14A), equipped with a DB-WAX capillary column (30 m long, 0.53 mm inner diameter, and 1 µm thickness) and a flame ionization detector. The detected liquid products were 1,2-propanediol (1,2-PDO), 1,3-propanediol (1,3-PDO), 1-propanol (1-PO), 2-propanol (2-PO), HA, and ethylene glycol (EG). For the quantification of each product, the pure chemical was purchased and a standard calibration curve with four concentration points and a coefficient of determination (R^2^) > 0.99 was made using GC. The activity of the catalysts was presented in terms of glycerol conversion (%), product selectivity (%), and product yield (%) as shown in Eqs. (1), (2), and (3), respectively:

1 $${\text{Glycerol}}\,{\text{conversion }}\left( \% \right) \, = \frac{{n_{{{\text{in}}}}^{{{\text{gly}}}} {-} n_{{{\text{out}}}}^{{{\text{gly}}}} }}{{n_{{{\text{in}}}}^{{{\text{gly}}}} }} \times 100$$

2 $${\text{Product}}\,{\text{selectivity }}\left( \% \right) \, = \frac{{n_{p} }}{{n_{{{\text{in}}}}^{{{\text{gly}}}} {-} n_{{{\text{out}}}}^{{{\text{gly}}}} }} \times \frac{{Z_{p} }}{{Z_{{{\text{gly}}}} }} \times 100$$

3 $${\text{Product}}\,{\text{yield }}\left( \% \right) \, = \frac{{\% {\text{ Glycerol}}\,{\text{conversion}} \times \% {\text{Product}}\,{\text{selectivity}} }}{100}$$

where ${\text{n}}_{{{\text{in}}}}^{{{\text{gly}}}}$ is the molar amount of glycerol before the reaction (blank), ${\text{n}}_{{{\text{out}}}}^{{{\text{gly}}}}$ is the molar amount of unreacted glycerol after the reaction, and ${\text{n}}_{{\text{p}}}$ is the molar amount of desired product. ${\text{Z}}_{{\text{p}}}$ and ${\text{Z}}_{{{\text{gly}}}}$ are the number of carbon atoms of the desired product and glycerol, respectively. Note that, for the catalytic activity data, “others” refers to 2-PO, HA, and other unquantified products including gaseous products (e.g. propane and ethane).

For the reusability test of the catalyst, after the first cycle of the reaction, the catalyst was separated from the liquid products using a centrifuge at 8000 rpm for 15 min. Then, the catalyst was washed three times with DI water, dried overnight in the hot-air oven, and reduced using the H_2_ flow as described previously before the new cycle of the reaction.

### Characterization of catalysts

The crystalline phases and the average metal crystallite sizes of the catalysts were analyzed using powder x-ray diffraction (XRD, JEOL JDX-3530 and Philips X-Pert) with Cu-Kα radiation at 45 kV and 40 mA at an angle (2θ) range of 10–80°. A step size of 0.02° and a step time of 0.5 s were used for the measurements.

The particle size distribution and the elemental composition mapping of the samples were analyzed using transmission electron microscopy with energy-dispersive X-ray spectroscopy (TEM with EDX: JEM-2100). After reduction with H_2_ at 350 °C, the samples were suspended in ethanol solvent, dropped on carbon film coated on Cu TEM grids, and dried in a chamber filled with N_2_ at room temperature before the analysis.

The surface area, pore volume, and average pore size of the catalysts were determined using an N_2_-physisorption analyzer (Brunauer–Emmett–Teller (BET): 3Flex Physisorption Micromeritics) at − 196 °C. Each catalyst was pretreated at 300 °C for 24 h in the system of the N_2_-physisorption analyzer before measurement. For each catalyst, the BET surface area was determined in a relative pressure (P/P^0^) range of 0.05–0.30; the total pore volume was determined at P/P^0^ of 0.995, and the pore size was computed using the Barrett-Joyner-Halenda method.

The elemental composition of the catalysts was determined using inductively coupled plasma-optical emission spectrometry (ICP-OES). Before the ICP-OES measurements, the solid samples were dissolved in hydrochloric acid solution.

The electronic states of cobalt (Co 2p) and copper (Cu 2p) for the samples were analyzed using x-ray photoelectron spectrometry (XPS; Kratos Axis Ultra DLD), using Al K_α_ for the x-ray source.

The reducibility of the catalysts was analyzed using H_2_-Temperature-Programmed Reduction (H_2_-TPR, Micromeritics AutoChem II). Before the H_2_-TPR experiments, each catalyst (0.1 g) was pretreated at 120 °C and a heating rate of 5 °C min^−1^ under an He flow (50 mL min^−1^). Then, it was cooled to 50 °C and then heated to 900 °C at a heating rate of 10 °C min^−1^ under a flow of 10% H_2_ in Ar. The H_2_ consumed during the reduction was continuously monitored using a thermal conductivity detector (TCD).

The acidity of the catalysts was analyzed using NH_3_ temperature-programmed desorption (NH_3_-TPD, Micromeritics AutoChem II). Before the NH_3_-TPD experiments, each catalyst (0.1 g) was pretreated using the same method as the pretreatment for the H_2_-TPR experiments. After the catalyst had cooled to 50 °C, it was exposed to 0.2% NH_3_ in He for 1 h followed by purging with He for 1 h. Finally, the NH_3_-TPD measurement was carried out from 50 to 900 °C at a heating rate of 10 °C min^-1^. The NH_3_-desorption was continuously monitored using a TCD. Note that all gas flow rates were 25 mL min^-1^ for gas/gas mixture measurements.

The surface morphology of the catalysts was analyzed using scanning electron microscopy (SEM; JEOL, JSM7600 F). Samples were imaged at a working distance of 4.0 mm and an acceleration voltage of 1.0 kV.

## Results and discussion

### Catalyst characterization

#### The crystallization analysis of catalysts by XRD

**Figure 1:**
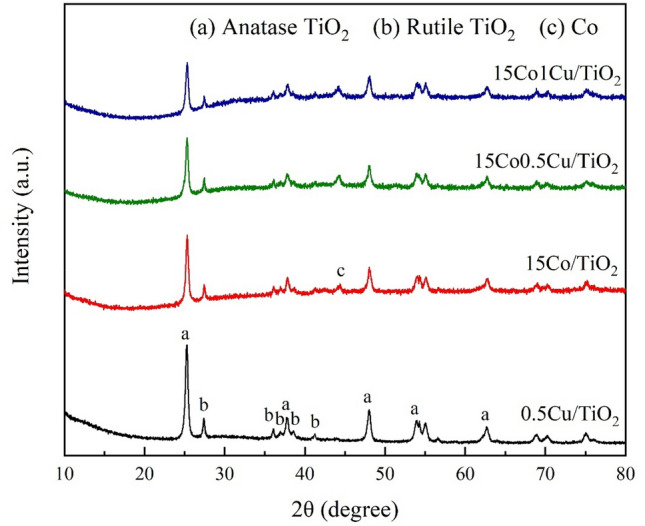
XRD patterns of (**a**) 0.5Cu/TiO_2_, (**b**) 15Co/TiO_2_, (**c**) 15Co0.5Cu/TiO_2_, and (**d**) 15Co1Cu/TiO_2_.

The XRD patterns of 15Co/TiO_2_, 15Co0.5Cu/TiO_2_, 15Co1Cu/TiO_2_, and 0.5Cu/TiO_2_ in the 2θ range from 10° to 80° are shown in Fig. 1. Note that the XRD measurement of all catalysts was carried out after the H_2_ reduction. As observed, the diffraction peaks of TiO_2_ with both anatase (2θ = 25.3°, 37.9°, 48.0°, 53.9°, and 62.6°)^17^ and rutile (2θ = 27.4°, 36.1°, 36.9°, 38.5°, and 41.2°)^16^ structures were observed in every catalyst, and all the XRD peaks of TiO_2_ were almost identical. This suggested that the loading of Co and Cu metal had not affected the crystallinity of the TiO_2_. In the XRD profiles of 15Co/TiO_2_, 15Co0.5Cu/TiO_2_, and 15Co1Cu/TiO_2_, only one clear diffraction peak of metallic Co at 2θ = 44.3°^18^ was observed; other diffraction peaks indicating the metallic Co (e.g. 2θ = 36.9° and 62.6°) were overlapped with the diffraction peaks of the TiO_2_. For the Cu-containing catalysts, no diffraction peak of Cu species was observed, possibly because the amount of Cu loading was small (< 2 wt%) and the crystal size of the Cu species was smaller than the size limit detection of the instrument (< 2.5 nm).

#### The morphological analysis of catalysts by TEM

**Figure 2:**
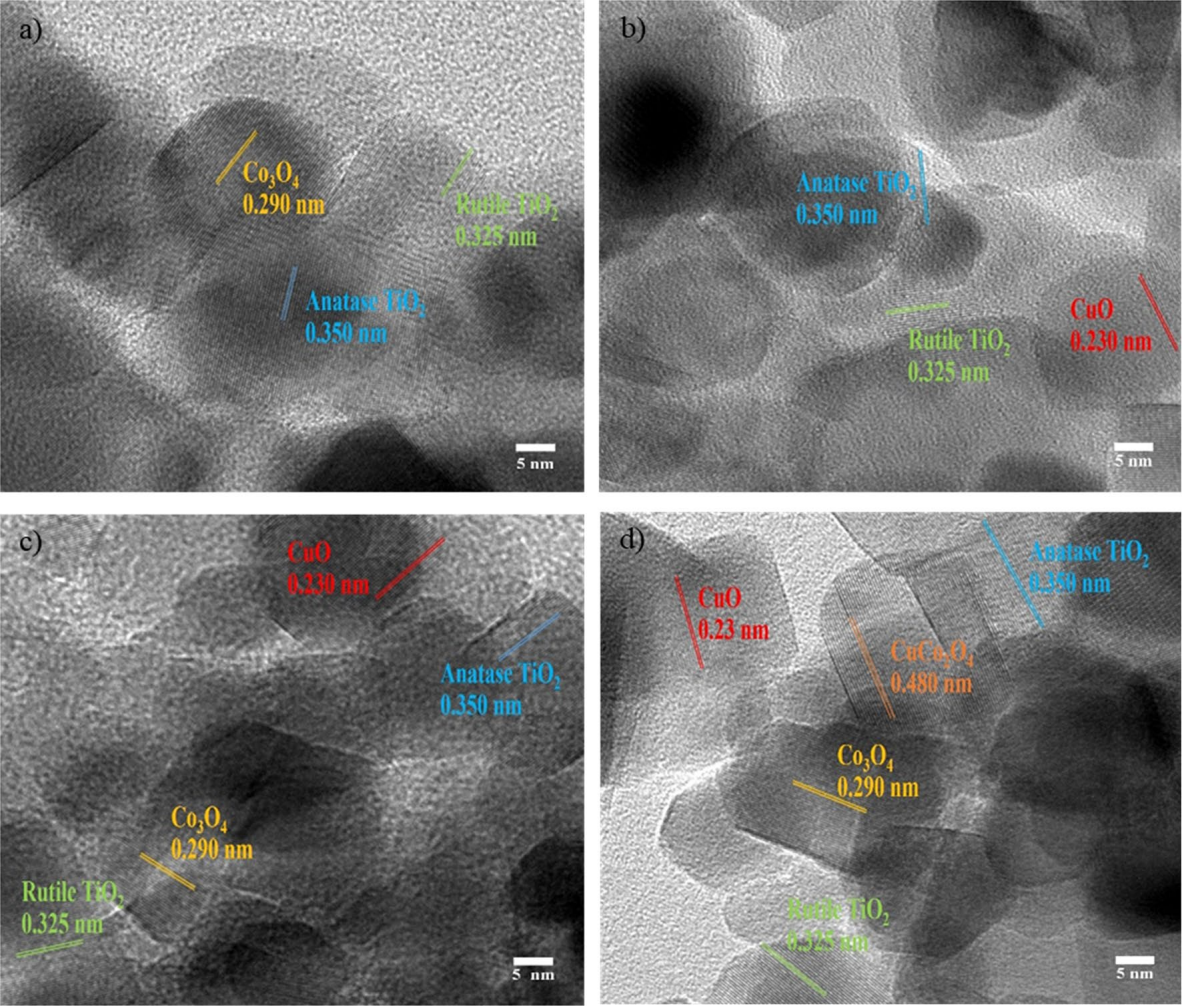
TEM images of (**a**) 15Co/TiO_2_, (**b**) 0.5Cu/TiO_2_, (**c**) 15Co0.5Cu/TiO_2_, and (**d**) 15Co1Cu/TiO_2_.

**Figure 3:**
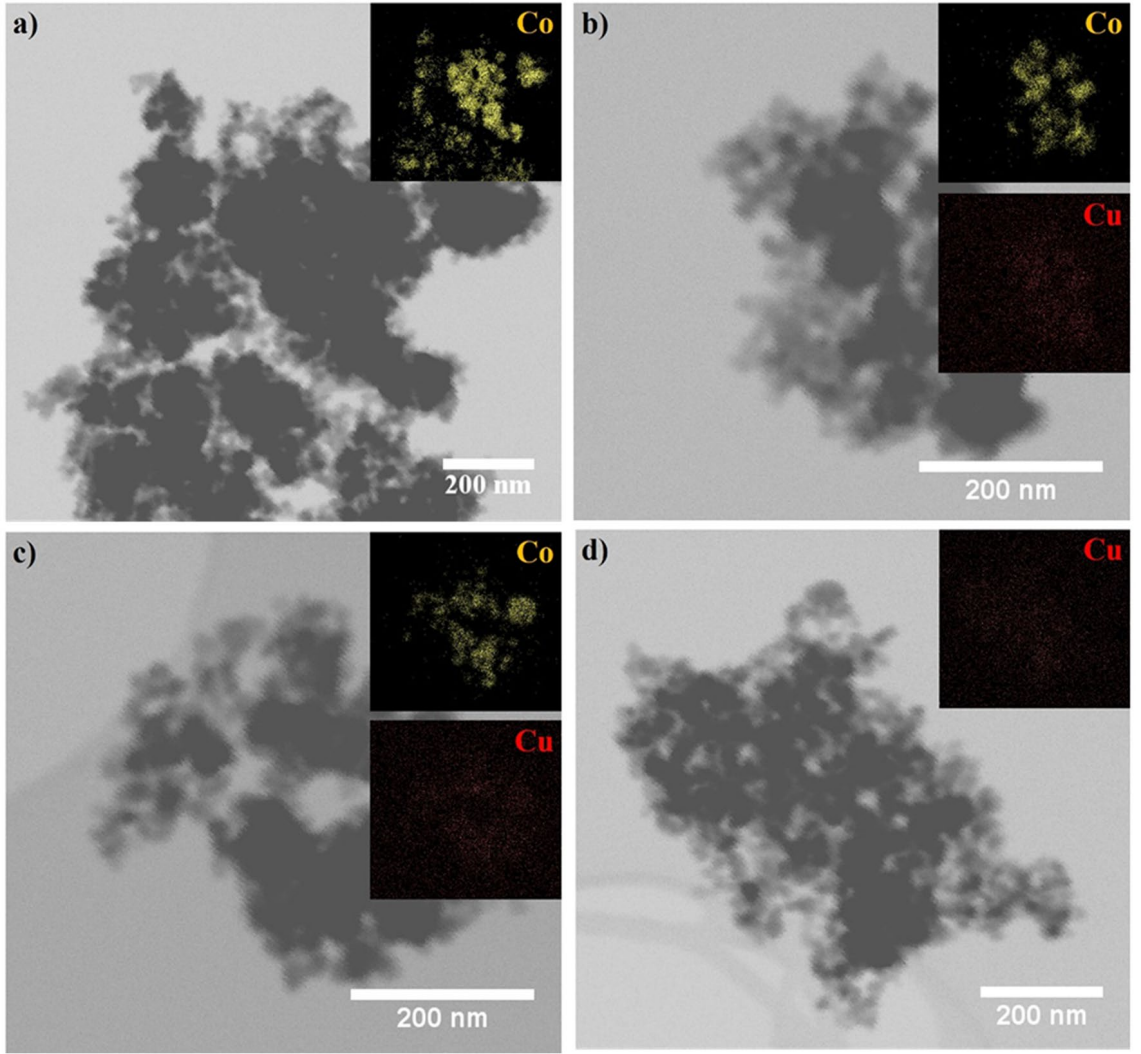
TEM with EDX images of (**a**) 15Co/TiO_2_, (**b**) 15Co0.5Cu/TiO_2_, (**c**) 15Co1Cu/TiO_2_, and (**d**) 0.5Cu/TiO_2_.

The structural morphologies of 15Co/TiO_2_, 15Co0.5Cu/TiO_2_, 15Co1Cu/TiO_2_, and 0.5Cu/TiO_2_ were characterized using TEM, as shown in Fig. 2. Note that the catalysts were exposed to air before imaging using TEM because of this limitation during the sample preparation for the TEM experiment. As observed, the oxides of Co and/or Cu were distributed throughout the TiO_2_ support. Every sample had a lattice d-spacing of 0.325 nm and 0.350 nm, specifying the crystal planes of the TiO_2_-rutile phase [1 1 0] and the TiO_2_-anatase phase [1 0 1], respectively^19^. Figure 2a illustrates the 15Co/TiO_2_ lattice d-spacing of 0.290 nm, indicating the crystal plane of Co_3_O_4_ [2 2 0]. Figure 2b illustrates the 0.5Cu/TiO_2_ lattice d-spacing of 0.230 nm, corresponding to the crystal plane of CuO [1 1 1]^20^. For the bimetallic catalysts in Figs. 2c and 2d, both crystal planes of Co_3_O_4_ and CuO were observed in each bimetallic catalyst. Additionally, 15Co1Cu/TiO_2_ had lattice d-spacing of 0.480 nm, assigned to the CuCO_2_O_4_ phase^21^. Furthermore, the elemental distributions on the surface of 15Co/TiO_2_, 15Co0.5Cu/TiO_2_, 15Co1Cu/TiO_2_, and 0.5Cu/TiO_2_ were analyzed using TEM with EDX-mapping, as shown in Fig. 3. The cobalt and copper species on the TiO_2_ support correlated to the bright yellow and red spots on the EDX-mapping images, respectively. As seen, both metal species were uniformly distributed on the surface of the support. Based on the XRD results, the Cu species in 0.5Cu/TiO_2_, 15Co0.5Cu/TiO_2_, and 15Co1Cu/TiO_2_, and the CuCO_2_O_4_ phase in 15Co1Cu/TiO_2_ were not observed. These results from TEM and TEM with EDX-mapping confirmed that the Cu and CuCo_2_O_4_ species were present in the catalysts.

#### The textural property analysis by N_2_-physisorption

**Figure 4:**
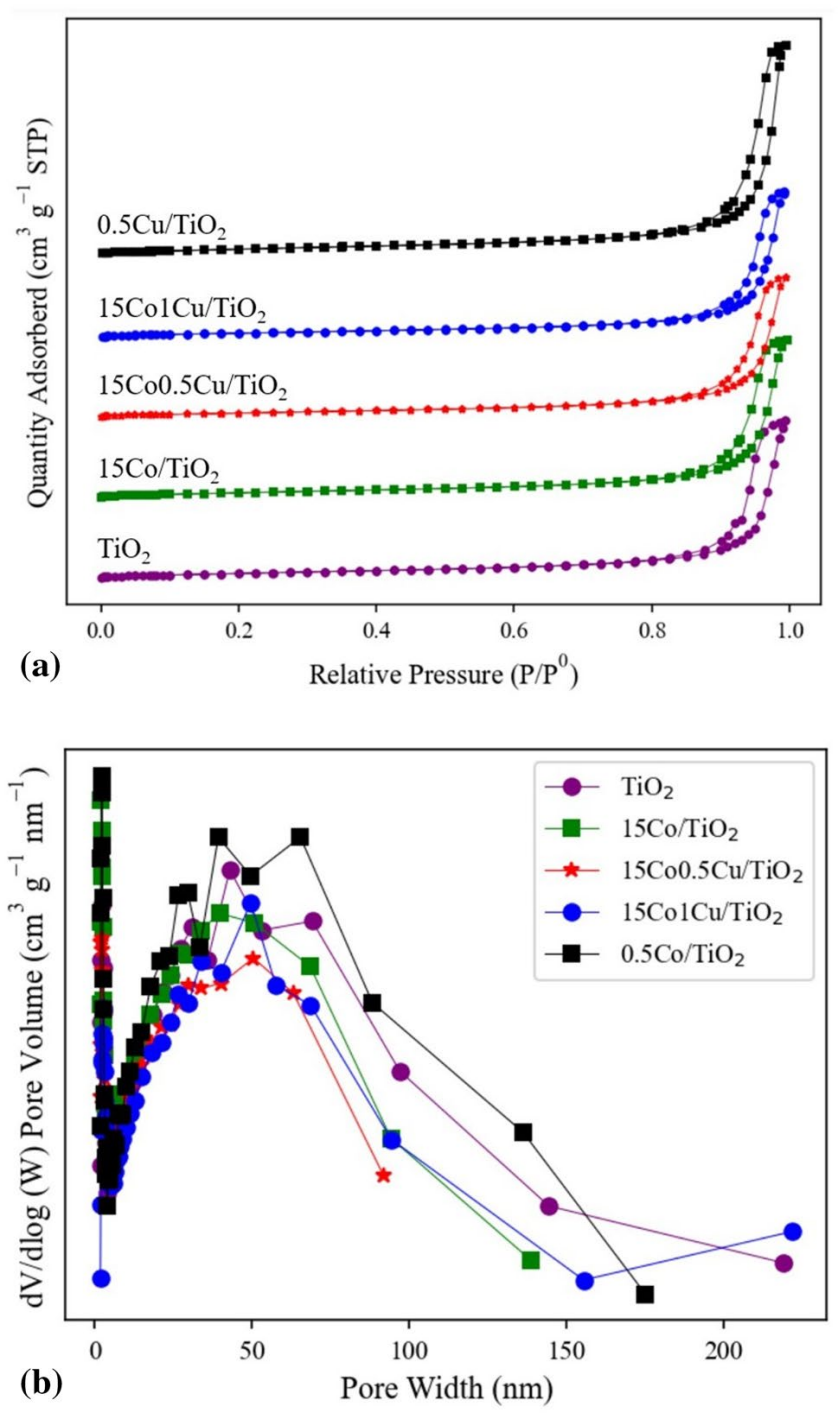
(**a**) N_2_ adsorption–desorption isotherms and (**b**) pore size distribution of TiO_2_, 15Co/TiO_2_, 15Co0.5Cu/TiO_2_, 15Co1Cu/TiO_2_, and 0.5Cu/TiO_2_.

The textural properties (BET surface area, pore size diameter, and pore volume) of TiO_2_, 15Co/TiO_2_, 15Co0.5Cu/TiO_2_, 15Co1Cu/TiO_2_, and 0.5Cu/TiO_2_ were determined using N_2_-sorption analysis, as shown in Table 1. Furthermore, to evaluate the N_2_ adsorption–desorption isotherm of each catalyst, the plot of the amount of N_2_ adsorbed and desorbed for each catalyst versus the relative pressure (P/P^0^) is shown in Fig. 4a. The plot of the pore size distribution of each catalyst is shown in Fig. 4b. Note that, before the BET measurement, the pure TiO_2_ was treated using the same procedure as the catalyst preparation, except for adding the active metals. In Table 1, the pure TiO_2_ support had the greatest BET surface area (50.5 m^2^ g^−1^) with the largest pore volume (0.69 cm^3^ g^−1^). After Co and/or Cu were impregnated onto the TiO_2_ support, the surface areas became smaller than those of the pure TiO_2_, and the pore volumes and average pore size diameters were similarly in the ranges 0.29–0.41 cm^3^ g^−1^ and 39.4–50.5 nm, respectively. Additionally, the greater the amount of loaded active metals, the lower the BET surface area, probably because the active metals filled the pores of the TiO_2_ support during the catalyst preparation.

**Table 1:** Textural properties and elemental composition of TiO_2_, 15Co/TiO_2_, 15Co0.5Cu/TiO_2_, 15Co1Cu/TiO_2_, and 0.5Cu/TiO_2_.

Catalysts	Metal composition (%)		BET surface area (m^2^ g^−1^)	Pore volume (cm^3^ g^−1^)	Average pore diameter (nm)
	Co	Cu			
TiO_2_	–	–	50.5	0.69	41.5
15Co/TiO_2_	14.45	–	41.8	0.31	40.0
15Co0.5Cu/TiO_2_	15.44	0.62	37.3	0.28	50.5
15Co1Cu/TiO_2_	14.83	1.22	34.1	0.29	49.6
0.5Cu/TiO_2_	–	0.44	48.9	0.41	39.4

The plots in Fig. 4a describe the adsorption–desorption behavior of N_2_ onto the catalyst surface. According to the IUPAC classification, all the catalysts were classified into the type IV isotherm, identifying them as a mesopore material consisting of multilayer adsorption followed by pore condensation^22^. A hysteresis loop can be seen above P/P^0^ values of 0.8 for every catalyst and it can be classified as type H3 hysteresis, revealing the capillary condensation of mesoporous materials^23^. For the pore size distribution curve shown in Fig. 4b, the peaks of all samples were very close to each other, approximately 50 nm.

#### The elemental composition analysis by ICP-OES

The elemental compositions of 15Co/TiO_2_, 15Co0.5Cu/TiO_2_, 15Co1Cu/TiO_2_, and 0.5Cu/TiO_2_ were determined using ICP-OES, as shown in Table 1. The results indicated that the actual amount of cobalt and/or copper in each catalyst was close to the theoretical value.

#### The oxidation states analysis of catalysts by XPS

**Figure 5:**
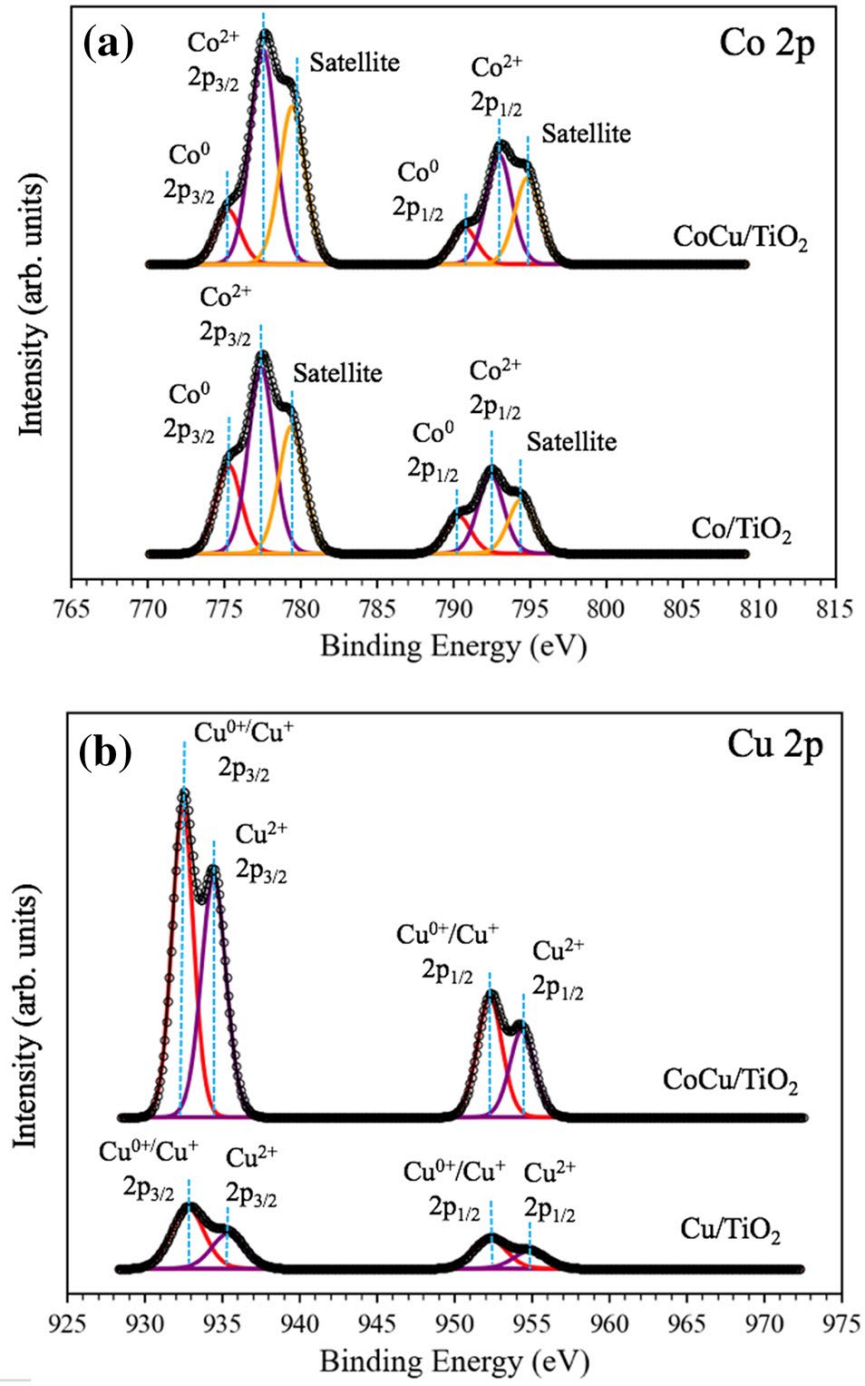
XPS spectra of (**a**) Co 2p of Co/TiO_2_ and CoCu/TiO_2_ and (**b**) Cu 2p of Cu/TiO_2_ and CoCu/TiO_2_.

The surface chemical states of the catalysts were determined using XPS, as shown in Fig. 5. For the Co spectra (Fig. 5a.), the deconvoluted peaks at 775.3, 777.4, and 779.4 eV in the orbital 2p_3/2_ position corresponded to the metallic Co^0^, Co^2+^, and satellites, respectively^24^. The binding energy (B.E.) value between each spin–orbit separation of Co 2p_3/2_ and Co 2p_1/2_ was consistent at 15 eV, which is characteristic of Co_3_O_4_ spinel^25^. The greatest area of the deconvoluted peak at 777.4 eV indicated that the Co^2+^ species represented the greatest amount among all the cobalt species^26^. For the Cu spectra (Fig. 5b), the deconvoluted peaks at 934.6 eV and 954.6 eV corresponded to Cu 2p_3/2_ and Cu 2p_1/2_, respectively. The splitting energy of 20 eV indicated the formation of Cu^2+^^27^. Furthermore, the deconvoluted peak at 932.5 eV in the orbital Cu 2p_3/2_ position could be assigned to Cu^0^ or Cu^+^ because the range of the B.E.s for Cu^0^ and Cu^+^ are overlapped^26^. After adding the Cu to Co/TiO_2_, a positive shift for the B.E. of Cu on CoCu/TiO_2_ was observed. This phenomenon was similarly observed in other Co–Cu catalysts for methanol decomposition to hydrogen production^26^ and direct synthesis of ethanol and higher alcohols from syngas^28^. This indicates that copper loses electrons and variation of copper chemical states in the bimetallic Co–Cu catalysts^26^. In other words, an electronic interaction occurs between the Co and Cu species over the TiO_2_ support.

#### The reducibility analysis of catalysts by H_2_-TPR

**Figure 6:**
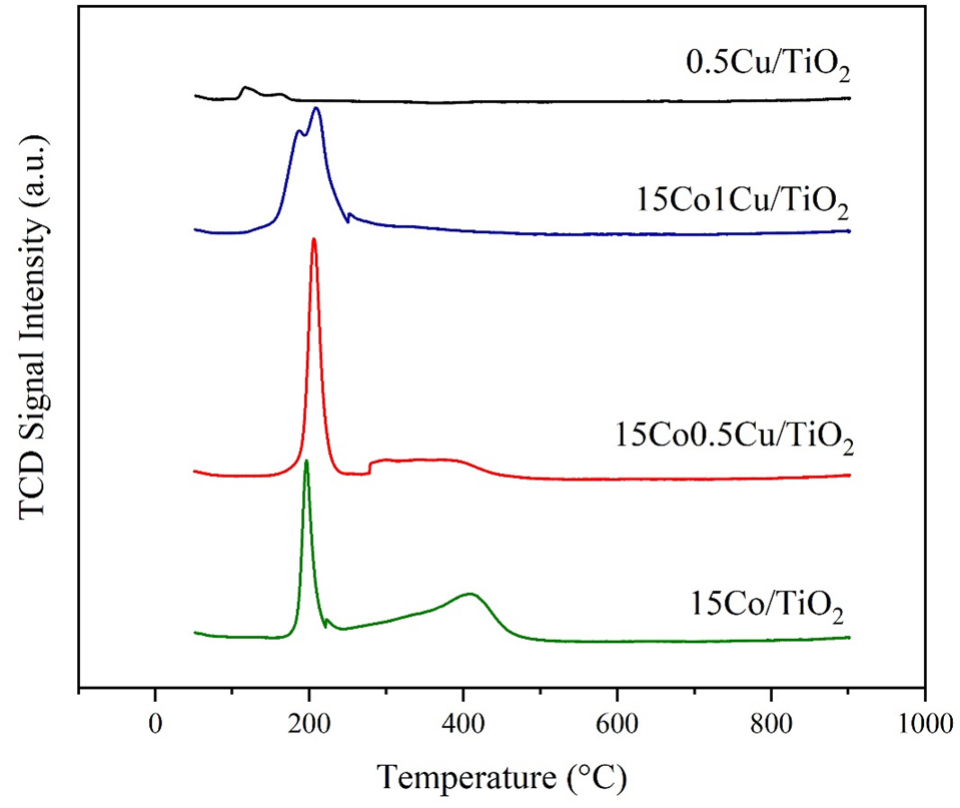
H_2_-TPR profiles of 15Co/TiO_2_, 15Co0.5Cu/TiO_2_, 15Co1Cu/TiO_2_, and 0.5Cu/TiO_2_.

The reducibility of 15Co/TiO_2_, 15Co0.5Cu/TiO_2_, 15Co1Cu/TiO_2_, and 0.5Cu/TiO_2_ was characterized using H_2_-TPR, as shown in Fig. 6. The H_2_-TPR profile of the catalysts can be used to identify the species of metal on the surface and the nature of the active site of the catalysts. For the monometallic catalysts, the 15Co/TiO_2_ had two reduction peaks, with one sharp peak at 200 °C and one broad reduction peak between 240 to 500 °C, assigned to the reductions of Co_3_O_4_ to CoO and CoO to Co, respectively^25^. The TPR profile of 0.5Cu/TiO_2_ had a reduction peak at 115 °C with a shoulder peak at 160 °C, corresponding to the reduction of Cu_2_O and CuO to Cu, respectively^29^. For the bimetallic catalysts, the TPR profile of 15Co0.5Cu/TiO_2_ had an overlapped peak at a lower temperature (205 °C) involved with the reduction of Co_3_O_4_ to CoO, CuO to Cu, and Cu_x_Co_3-x_O_4_ to Cu and Co^30^, and a relatively large broad reduction peak at higher temperature (approximately 280–500 °C), assigned to the reduction of CoO to Co^31^. Interestingly, 15Co1Cu/TiO_2_ had an overlapped reduction peak around 100–250 °C with a relatively small broad peak around 250–300 °C. This can be explained by 15Co1Cu/TiO_2_, with the main peak and a shoulder peak around 100–250 °C being attributed to a complicated reduction of the oxides of Cu and Co to Cu and Co, suggesting that the Cu- and Co-species particles were in close contact, which led to an occurrence of a spillover mechanism during the reduction of the Co–Cu catalysts^32^. In other words, because of the close contact of the Cu- and Co-species particles, the adsorbed atomic hydrogen could transfer from Cu^0^ to Co oxide species and encouraged reduction to Co^0^.

#### The acidity analysis of the catalysts by NH_3_-TPD

The acidity of 15Co/TiO_2_, 15Co0.5Cu/TiO_2_, and 0.5Cu/TiO_2_ was determined using NH_3_-TPD, as shown in Table 2. The acid sites were calculated from the peak areas of NH_3_ desorption signals in different temperature ranges, which can be defined as weak acid sites (< 300 °C), medium acid sites (300–500 °C), and strong acid sites (> 500 °C)^32^. From Table 2, the TiO_2_ support had the highest number of total acid sites. After the metals were impregnated, the total acid sites of catalysts decreased, which was attributed to the occupation of acid sites by metal species^11^. For the bimetallic catalyst, the addition of Cu to Co/TiO_2_ enhanced the formation of acid sites due to the formation of CuCo_2_O_4_ species^32^ (as deduced from TEM) and the synergistic interaction of these metals with the TiO_2_ support^11^.

**Table 2:** Acidity of TiO_2_, 15Co/TiO_2_, 15Co0.5Cu/TiO_2_, and 0.5Cu/TiO_2_.

Catalysts	Acid amount (mmol of NH_3_/g of catalyst)			
	Weak acid	Moderate acid	Strong acid	Total acid
TiO_2_	0.23	0.10	0.15	0.48
15Co/TiO_2_	0.08	0.11	0.06	0.25
15Co0.5Cu/TiO_2_	0.12	0.10	0.06	0.28
0.5Cu/TiO_2_	0.08	0.04	0.07	0.19

### The activity of catalysts for hydrogenolysis of glycerol to 1,2-PDO

The prepared catalysts were investigated for hydrogenolysis of glycerol to 1,2-PDO at 230 °C with a hydrogen pressure of 4 MPa for 4 h. The results in terms of glycerol conversion, selectivity, and 1,2-PDO yield are summarized in Table 3. The 15Co/TiO_2_ monometallic catalyst achieved 46.2% of glycerol conversion and 81.1% of 1,2-PDO selectivity, giving a 1,2-PDO yield of 37.5%; meanwhile, the 0.5Cu/TiO_2_ monometallic catalyst had a very low 1,2-PDO yield at 1.0%. Increasing the Cu loading on Co/TiO_2_ increased the 1,2-PDO formation, yielding the maximum 1,2-PDO at 51.8% with 60.2% glycerol conversion and 86.0% of 1,2-PDO selectivity when the Cu loading was 0.5 wt%. This was because i) there was a synergistic catalytic effect between the Co and Cu that occurred between the interfaces of these metal particles; ii) there was an increase in acid sites^33^, especially the weak acid site when the Cu was added to Co/TiO_2_ (as indicated in the NH_3_-TPD results in Table 2) that can favor the activation of the C—O bond from glycerol to HA during the dehydration step^34^ so that the glycerol conversion increased; and iii) the presence of multiple Co sites (as indicated in the results of XPS in Fig. 5 and H2-TPR in Fig. 6) promoted the overall hydrogenolysis of glycerol to 1,2-PDO^21,32^.

**Table 3:** Catalytic activity for hydrogenolysis of glycerol over CoCu/TiO_2_.

Catalyst	Conversion (%)	Selectivity (%)					1,2-PDO yield (%)
		1,2-PDO	1,3-PDO	EG	1-PO	Others	
15Co/TiO_2_	46.2	81.1	1.6	7.7	4.7	0.8	37.5
15Co0.25Cu/TiO_2_	47.4	84.1	1.3	5.8	5.3	1.0	39.9
15Co0.5Cu/TiO_2_	60.2	86.0	1.3	7.0	3.6	0.8	51.8
15Co1Cu/TiO_2_	44.5	90.5	0.9	3.5	3.2	1.1	40.3
15Co2Cu/TiO_2_	30.3	85.1	1.0	1.7	8.7	2.4	25.8
0.5Cu/TiO_2_	2.3	41.6	14.8	2.0	22.2	17.8	1.0

Reaction conditions: 0.5 g catalyst, 230 °C, 4 MPa, and 4 h.

Increasing the Cu loading on Co/TiO_2_ from 0.5 to 2 wt% decreased the performance of the catalysts, probably because the excess amount of Cu over the surface (as indicated by the H_2_-TPR results in Fig. 6) could reduce the number of interfaces between Co and Cu. In addition, the catalyst’s pores could have been blocked by the high metal loadings^35^ (as indicated by the BET results in Table 1).

### Effect of operating conditions

#### Catalyst-to-glycerol ratio

**Figure 7:**
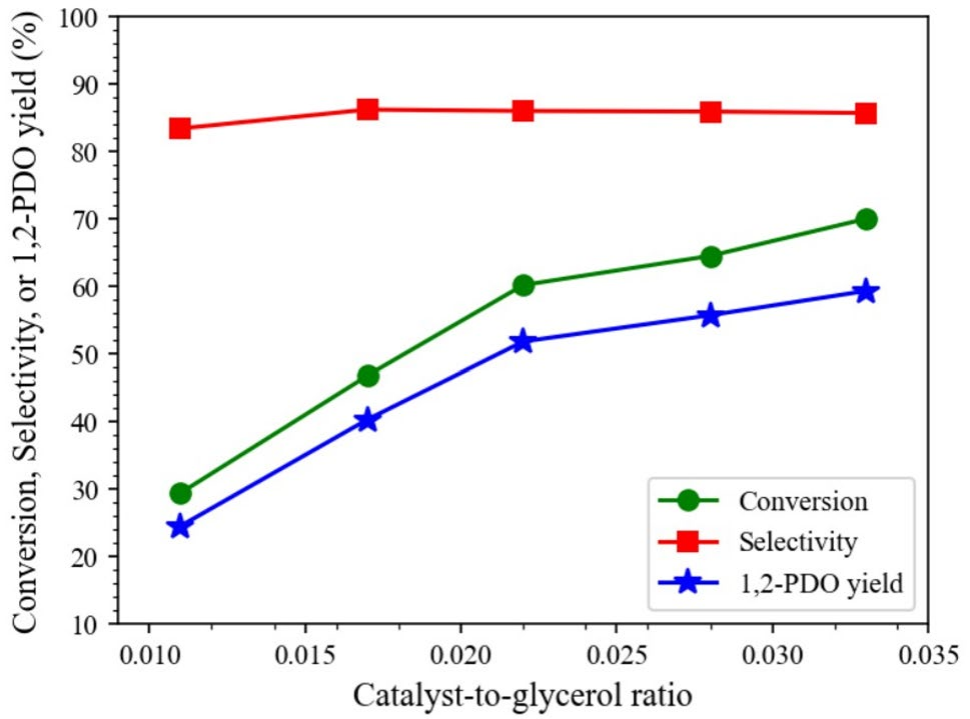
Effect of catalyst-to-glycerol ratio on hydrogenolysis of glycerol, with reaction conditions: 230 °C, 4 MPa, and 4 h, and glycerol solution amount fixed at 20 mL (22.6 g).

The effect of the glycerol-to-catalyst ratio on the hydrogenolysis of glycerol over 15Co0.5Cu/TiO_2_ is presented in Fig. 7. The catalyst-to-glycerol ratio (on a weight basis) was varied from 0.011 to 0.033. As observed, increasing the catalyst-to-glycerol ratio from 0.011 to 0.033 increased the glycerol conversion and the 1,2-PDO yield from 29.4% to 69.8% and 24.5% to 59.3%, respectively, because the number of active sites increased with the increasing catalyst-to-glycerol ratio ^33,36^. It was also observed that the selectivity of 1,2-PDO did not change much in this range for the catalyst-to-glycerol ratio; the selectivity of 1,2-PDO was approximately 83–86%, implying that over hydrogenolysis of 1,2-PDO did not occur.

#### Reaction temperature

**Figure 8:**
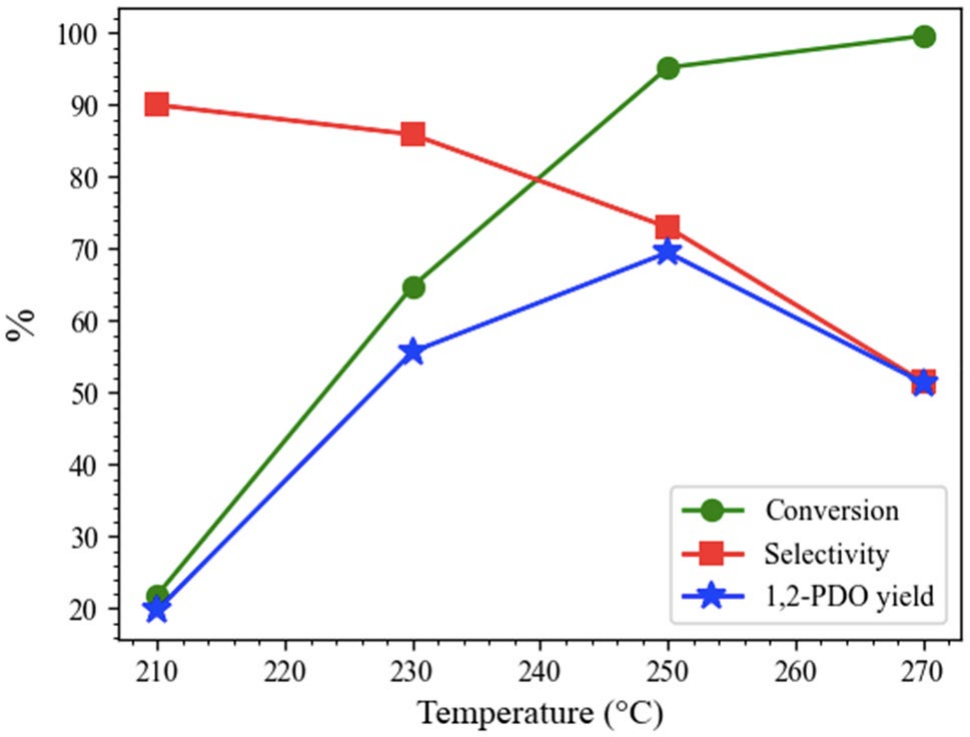
Effect of reaction temperature on hydrogenolysis of glycerol, with reaction conditions: catalyst-to-glycerol ratio of 0.028, 4 MPa, and 4 h, and glycerol solution amount fixed at 20 mL (22.6 g).

The effect was investigated of the reaction temperature on the activity of the hydrogenolysis of glycerol over 15Co0.5Cu/TiO_2_ and the results are shown in Fig. 8. The increase in reaction temperature from 210 to 270 °C led to a large increase in the glycerol conversion from 21.9 to 99.6%. The selectivity toward 1,2-PDO gradually decreased from 90.0 to 51.5% and the maximum 1,2-PDO yield at 69.5% was obtained at 250 °C. The results indicated that the higher reaction temperature favored glycerol conversion but led to lower selectivity of 1,2-PDO due to the formation of 1-PO and 2-PO^11,13^.

#### Reaction pressure

**Figure 9:**
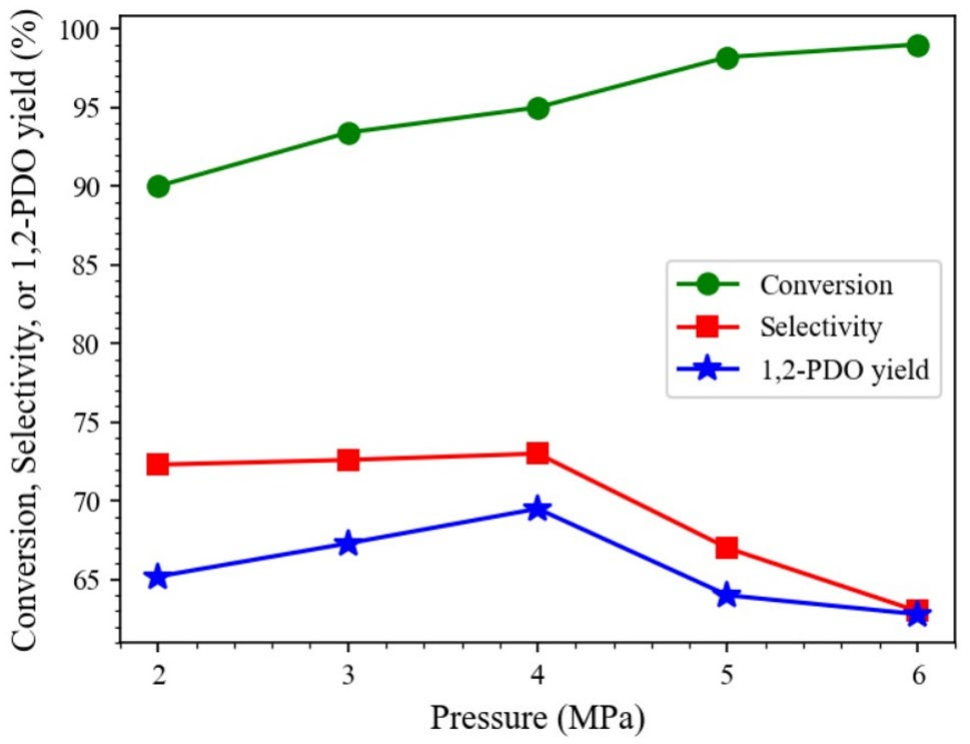
Effect of hydrogen pressure on hydrogenolysis of glycerol, with reaction conditions: 0.028 catalyst-to-glycerol ratio, 250 °C, and 4 h, and glycerol solution amount fixed at 20 mL (22.6 g).

The effect was examined of initial hydrogen pressure in the pressure range 2–6 MPa on the glycerol hydrogenolysis over 15Co0.5Cu/TiO_2_ at 250 °C, as presented in Fig. 9. Increasing the initial pressure from 2 to 6 MPa resulted in an increased glycerol conversion from 90.2 to 99.8%, because the solubility of hydrogen into an aqueous solution was enhanced by the increased pressure^13^. The selectivity toward 1,2-PDO slightly increased from 72.3 to 73.0% and further sharply decreased to 63.0% at 6 MPa hydrogen pressure due to the degradation of 1,2-PDO to 1-PO and 2-PO^14^. Therefore, the optimal initial hydrogen pressure to obtain the maximum 1,2-PDO yield was 4 MPa for this catalyst.

#### Reaction time

**Figure 10:**
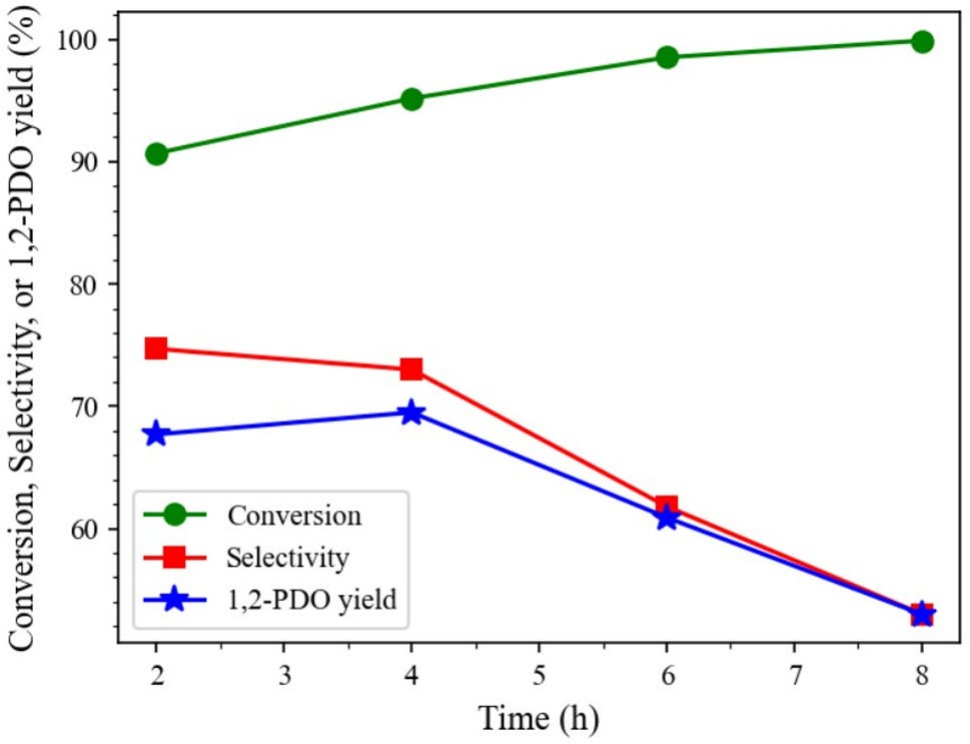
Effect of reaction time on hydrogenolysis of glycerol, with reaction conditions: 0.028 catalyst-to-glycerol ratio, 250 °C, and 4 MPa, and glycerol solution amount fixed at 20 ml (22.6 g).

The effect of reaction time on the glycerol hydrogenolysis over 15Co0.5Cu/TiO_2_ was investigated at a catalyst-to-glycerol ratio of 0.028, a reaction temperature of 250 °C, and an initial hydrogen pressure of 4 MPa, as shown in Fig. 10. As seen, the glycerol conversion increased from 90.2 to 99.8% when the reaction time increased from 2 to 8 h. However, the selectivity of 1,2-PDO decreased from 72.3 to 63.0% with increasing reaction time, which also contributed to the degradation of formed 1,2-PDO to 1-PO^7^. Overall, the local optimal reactions to obtain the maximum 1,2-PDO yield (69.5%) was a catalyst-to-glycerol ratio of 0.028, a reaction temperature of 250 °C, an initial hydrogen pressure of 4 MPa, and a reaction time of 4 h for this catalyst.

### Reusability test of 15Co0.5Cu/TiO_2_

**Figure 11:**
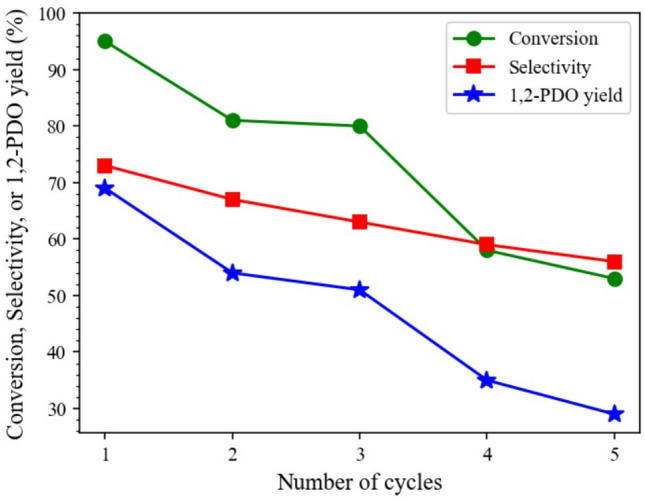
Reusability of 15Co0.5Cu/TiO_2_ for hydrogenolysis of glycerol to 1,2-PDO, with reaction conditions: 0.028 catalyst-to-glycerol ratio, 250 °C, and 4 MPa, and glycerol solution amount fixed at 20 ml (22.6 g).

**Figure 12:**
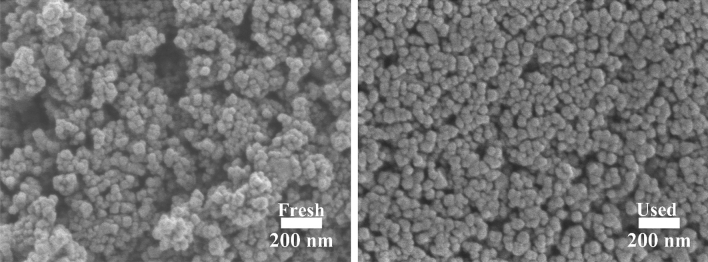
SEM images of fresh and used 15Co0.5Cu/TiO_2_.

**Figure 13:**
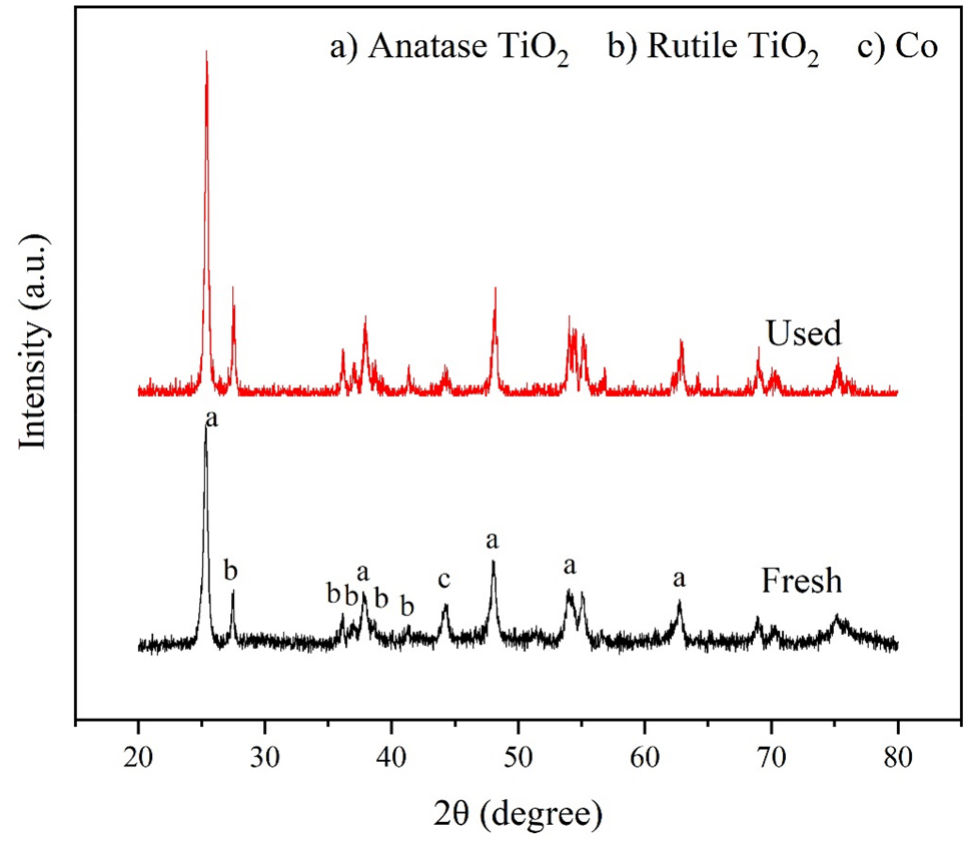
XRD pattern of fresh and used 15Co0.5Cu/TiO_2_.

The reusability of the catalyst is an important factor that should be considered for commercial purposes. The reusability of 15Co0.5Cu/TiO_2_ for the hydrogenolysis of glycerol over five cycles is shown in Fig. 11. As observed, the glycerol conversion and the 1,2-PDO yield decreased after each hydrogenolysis of glycerol reaction. After five cycles, the glycerol conversion and 1,2-PDO yields decreased from 95.2% and 69.5% to 52.5% and 29.3%, respectively, while 1,2-PDO selectivity gradually decreased from 73.0% to 55.8%. The catalyst used for five cycles was further analyzed for its physicochemical properties using SEM, XRD, and N_2_-physisorption, as shown in Figs. 12 and 13, and Table 4, respectively. Comparing the SEM images, N_2_-physisorption results, and XRD pattern of the fresh and used catalysts, the surface morphology, the textural properties, and the crystalline properties were similar. In contrast, the analysis of the fresh catalyst and the used catalyst after five cycles using ICP-OES (see Table 5) showed that the amount of Co had substantially decreased in the used catalyst. Furthermore, the crude product of the hydrogenolysis of the glycerol reaction was analyzed using ICP-OES; it was found that the amounts of Co and Cu in the crude product were 374.5 and 0.2 ppm, respectively. These results indicated that gradual leaching, especially of Co, occurred during the reaction and the recycling process, leading to the gradual deactivation of the catalyst, similar to the results reported by Feng et al.^7^ A further study in the prevention of the leaching is, therefore, needed.

**Table 4:** Textural properties of fresh and used 15Co0.5Cu/TiO_2_.

Catalyst	BET surface area (m^2^ g^−1^)	Pore volume (cm^3^ g^−1^)	Average pore diameter (nm)
Fresh 15Co0.5Cu/TiO_2_	37.3	0.28	50.5
Used 15Co0.5Cu/TiO_2_	37.7	0.33	65.5

**Table 5:** Elemental composition of fresh and used 15Co0.5Cu/TiO_2_.

Catalysts	Metal composition (%)	
	Co	Cu
Fresh 15Co0.5Cu/TiO_2_	15.44	0.62
Used 15Co0.5Cu/TiO_2_	11.10	0.77

### Comparative activity of the catalysts for hydrogenolysis of glycerol to 1,2-PDO

**Figure 14:**
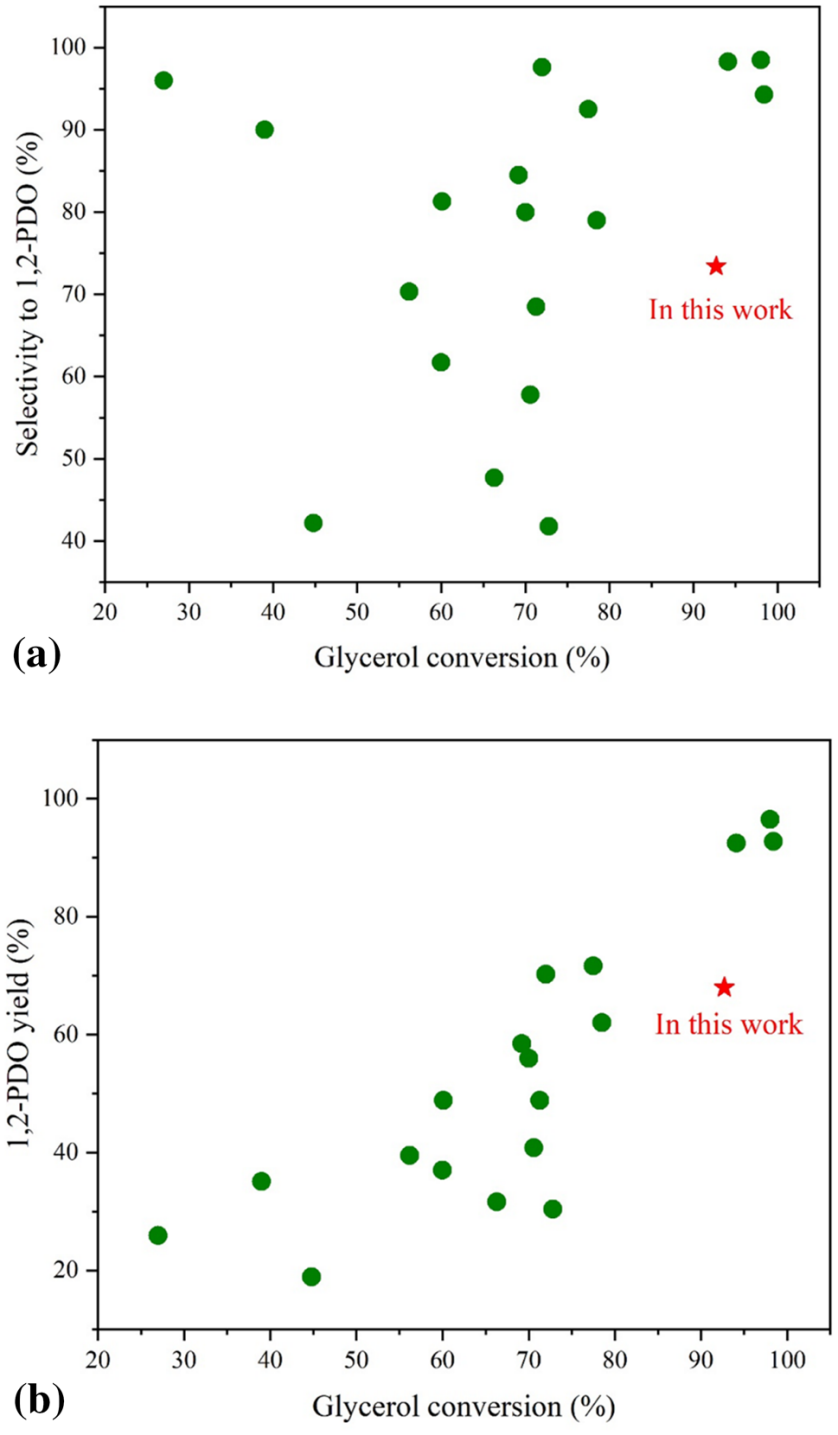
Comparative activity of Co- or Cu-based catalysts for hydrogenolysis of glycerol (**a**) selectivity to 1,2-PDO versus glycerol conversion and (**b**) 1,2-PDO yield versus glycerol conversion.

From previous research, most catalysts were bifunctional, consisting of acidic sites and metallic sites, which are involved in the dehydration and hydrogenation steps, respectively. Figure 14 illustrates the comparative activity of bifunctional catalysts for the hydrogenolysis of glycerol to 1,2-PDO, with the detail of each catalyst shown in Table S1. High reaction temperatures around 170–250 °C are commonly used since the hydrogenolysis of glycerol is an endothermic reaction. In addition, high initial H_2_ pressures around 1–6 MPa are preferred because they provide an extensive massive dispersion of H_2_ and more H_2_ is dissolved in the liquid phase, which is advantageous for the hydrogenolysis of glycerol. Comparing our catalyst performance with others, CoCu/TiO_2_ was highly effective in the hydrogenolysis of glycerol to 1,2-PDO, achieving a maximum 1,2-PDO yield at 69.5% with 95.2% glycerol conversion in the current study. Catalysts providing a 1,2-PDO yield above 90% are shown in Fig. 14 include Cu–Zn-Mg–Al-O with the addition of NaOH^37^, Cu@SiO_2_ core–shell-catalysts with a Cu/Si atomic ratio of 2^38^, and PdCu-KF/Al_2_O_3_^7^. Nevertheless, our catalyst can be easily prepared, the Cu and Co precursors are relatively inexpensive, and the activity of CoCu/TiO_2_ can be further improved by fine-tuning the Co-to-Cu-to-TiO_2_ ratio and optimizing the product formation using design of experiments.

## Conclusion

The 15Co0.5Cu/TiO_2_ catalyst was highly active for the hydrogenolysis of glycerol to 1,2-PDO and has potential for industrial use. The maximum 1,2-PDO yield was achieved at 69.5% with 95.2% glycerol conversion and 73.0% 1,2-PDO selectivity under the maximized conditions of a catalyst-to-glycerol ratio of 0.028 and a reaction temperature of 250 °C at 4 MPa H_2_ for 4 h. The addition of Cu to Co/TiO_2_ caused a synergistic catalytic effect between the Co and Cu, providing much higher activity for 1,2-PDO formation than from the monometallic catalysts. The NH_3_-TPD and H_2_-TPR analyses suggested that an increase in the number of acid sites (especially weak acid sites) and the presence of multiple Co sites, respectively, can favor the hydrogenolysis of glycerol to 1,2-PDO. In the study on the effects of operating conditions, increasing the reaction temperature, initial pressure, and reaction time increased the glycerol conversion but decreased the selectivity to 1,2-PDO due to the degradation of formed 1,2-PDO to lower alcohols (1-propanol and 2-propanol).

## Supplementary Information

Supplementary Information.
