# Supplementary material for: Highly efficient TiO2-supported Co–Cu catalysts for conversion of glycerol to 1,2-propanediol
Source: Sci Rep. 2021 Nov 29;11:23042. doi: 10.1038/s41598-021-02416-7 (PMC8630069; doi:10.1038/s41598-021-02416-7)
Supplement: Supplementary file 1 — Supplementary Information. [file 41598_2021_2416_MOESM1_ESM.pdf]

Supplementary data for:

Highly efficient TiO_2-supported Co-Cu catalysts for conversion of glycerol to 1,2-propanediol

Wongsaphat Mondach[1,2]*, Sarun Chanklang* [1]*, Pooripong Somchuea* [1]*, Thongthai Witoon*[1,2,3]*, Metta Chareonpanich*[1,2,3]*, Kajornsak Faungnawakij*[4]*, Hiesang Sohn*[5] *Anusorn Seubsai*[1,2,3]*

[1] *Department of Chemical Engineering, Faculty of Engineering, Kasetsart University, Bangkok 10900, Thailand*

[2] *Center of Excellence on Petrochemical and Materials Technology, Kasetsart University, Bangkok 10900, Thailand*

[3] *Research Network of NANOTEC–KU on NanoCatalysts and NanoMaterials for Sustainable Energy and Environment, Kasetsart University, Bangkok 10900, Thailand*

[4] *Nanomaterials for Energy and Catalysis Laboratory, National Nanotechnology Center (NANOTEC), National Science and Technology Development Agency (NSTDA), Klong Laung, Pathumthani 12120, Thailand*

[5] *Department of Chemical Engineering, Kwangwoon University, Seoul 01897, Korea*

***Corresponding author**: fengasn@ku.ac.th

Supplementary Table S1. Comparative activity of Co- or Cu-based catalysts for hydrogenolysis of glycerol.

Catalysts	Conditions	Glycerol conversion (%)	1,2-PDO yield (%)	Ref.
Ru-Co/ZrO_2	180 °C, 5 MPa, 10 h, 50 mg catalyst	56.2	39.5	1
Ru/TiO_2	170 °C, 3 MPa, 12 h, 102 mg catalyst	66.3	31.6	2
RuCo/TiO_2	180 °C, 5 MPa, 10h, 50 mg catalyst	72.8	30.4	3
Co/LDHs	200 °C, 2.0 MPa, 12 h, 0.3 g catalyst	70.6	40.8	4
Co/MgO	200 °C, 2.0 MPa, 0.2 g catalyst, 9 h	44.8	18.9	5
Ce-NiCo/Al_2O_3	220 °C, 6.0 MPa, 10 h, 2.0 g catalyst	71.3	48.8	6
RuCu/TiO_2	200 °C, 2.5 MPa, 0.603 g cat., 12 h	39.0	35.1	7
Cu/SiO_2	200 °C, 3.5 MPa, 10 h, 1 g catalyst	98.0	96.5	8
Co/ZnO	180 °C, 4.0 MPa, 0.6 g, 8 h	70.0	56.0	9

Ru/HY	220 °C, 3 MPa, 10 h	60.1	48.9	10
PdCu-KF/Al_2O_3	200 °C, 2.5 MPa, 20 h.	94.1	92.5	11
Cu/Boehmite	200 °C, 4.0 MPa, 6 h	77.5	71.7	12
CuAg/Al_2O_3	200 °C, 3.6 MPa, 10 h	27.0	25.9	13
Cu/Dol	200 °C, 4 MPa, 10h	78.5	62.0	14
Nb/Pd-Zr-Al	200 °C, 3.5 MPa, 8 h	69.2	58.5	15
Cu-Zn-Mg-Al-O	210 °C, 4.5 MPa, 12h, 1 g NaOH	98.4	92.8	16
Cu/MgO	180 °C, 3.0 MPa, 20 h	72.0	70.3	17
Copper chromite	300 °C, 1.0 MPa, 4 h	60.0	37.0	18

References

1 Feng, J., Zhang, Y., Xiong, W., Ding, H. & He, B. Hydrogenolysis of glycerol to 1,2-propanediol and ethylene glycol over Ru-Co/ZrO_2 catalysts. *Catalysts* **6**, 51 (2016).

2 Feng, J. *et al.* Hydrogenolysis of glycerol to glycols over ruthenium catalysts: Effect of support and catalyst reduction temperature. *Catal. Commun.* **9**, 1458-1464 (2008).

3 Feng, J., Xu, B., Liu, D. R., Xiong, W. & Wang, J. B. Performances of titania-supported Ru-based bimetallic catalysts in glycerol hydrogenolysis reaction. *Adv. Mat. Res.* **791-793**, 12-15 (2013).

4 Guo, X., Li, Y., Song, W. & Shen, W. Glycerol hydrogenolysis over Co catalysts derived from a layered double hydroxide precursor. *Catal. Lett.* **141**, 1458-1463 (2011).

5 Guo, X. *et al.* Co/MgO catalysts for hydrogenolysis of glycerol to 1,2-propanediol. *Appl. Catal. A: Gen.* **371**, 108-113 (2009).

6 Jiang, T., Kong, D., Xu, K. & Cao, F. Hydrogenolysis of glycerol aqueous solution to glycols over Ni–Co bimetallic catalyst: effect of ceria promoting. *Appl. Petrochem. Res.* **6**, 135-144 (2015).

7 Salazar, J. B. *et al.* Selective production of 1,2-propanediol by hydrogenolysis of glycerol over bimetallic Ru–Cu nanoparticles supported on TiO_2. *Appl. Catal. A: Gen.* **482**, 137-144 (2014).

8 Li, K. T., Wang, C. H. & Wang, H. C. Hydrogenolysis of glycerol to 1,2-propanediol on copper core-porous silica shell-nanoparticles. *J. Taiwan Inst. Chem. Eng.* **52**, 79-84 (2015).

9 Rekha, V., Sumana, C., Douglas, S. P. & Lingaiah, N. Understanding the role of Co in Co–ZnO mixed oxide catalysts for the selective hydrogenolysis of glycerol. *Appl. Catal. A: Gen.* **491**, 155-162 (2015).

10 Jin, S., Xiao, Z., Li, C., Williams, C. T. & Liang, C. Hydrogenolysis of glycerol over HY zeolite supported Ru catalysts. *J. Energy Chem.* **23**, 185-192 (2014).

11 Feng, Y. S. *et al.* Selective hydrogenolysis of glycerol to 1,2-propanediol catalyzed by supported bimetallic PdCu-KF/γ-Al_2O_3. *Chem. Eng. J.* **281**, 96-101 (2015).

12 Wu, G., Guan, N. & Li, L. Low temperature CO oxidation on Cu–Cu_2O/TiO_2 catalyst prepared by photodeposition. *Catal. Sci. Technol.* **1**, 601-608 (2011).

13 Zhou, J., Guo, L., Guo, X., Mao, J. & Zhang, S. Selective hydrogenolysis of glycerol to propanediols on supported Cu-containing bimetallic catalysts. *Green Chem.* **12**, 1835-1843 (2010).

14 Azri, N., Irmawati, R., Nda-Umar, U. I., Saiman, M. I. & Taufiq-Yap, Y. H. Effect of different supports for copper as catalysts on glycerol hydrogenolysis to 1,2-propanediol. *J. King Saud Univ. Sci.* **33**, 101417 (2021).

15 Cai, F., Jin, F., Hao, J. & Xiao, G. Selective hydrogenolysis of glycerol to 1,2-propanediol on Nb-modified Pd−Zr−Al catalysts. *Catal. Commun.* **131**, 105801 (2019).

16 Mondal, S., Janardhan, R., Meena, M. L. & Biswas, P. Highly active Cu-Zn-Mg-Al-O catalyst derived from layered double hydroxides (LDHs) precursor for selective hydrogenolysis of glycerol to 1,2-propanediol. *J. Environ. Chem. Eng.* **5**, 5695-5706 (2017).

17 Yuan, Z. *et al.* Biodiesel derived glycerol hydrogenolysis to 1,2-propanediol on Cu/MgO catalysts. *Bioresour. Technol.* **101**, 7099-7103 (2010).

18 Marinoiu, A. *et al.* Hydrogenolysis of glycerol to propylene glycol using heterogeneous catalysts in basic aqueous solutions. *React. Kinet. Mech. Catal.* **110**, 63-73 (2013).
